# Single fluoxetine treatment before but not after stress prevents stress-induced hippocampal long-term depression and spatial memory retrieval impairment in rats

**DOI:** 10.1038/srep12667

**Published:** 2015-07-28

**Authors:** Huili Han, Chunfang Dai, Zhifang Dong

**Affiliations:** 1 Ministry of Education Key Laboratory of Child Development and Disorders, Children’s Hospital of Chongqing Medical University, Chongqing 400014, PR China; 2 Chongqing Key Laboratory of Translational Medical Research in Cognitive Development and Learning and Memory Disorders, Children’s Hospital of Chongqing Medical University, Chongqing 400014, PR China

## Abstract

A growing body of evidence has shown that chronic treatment with fluoxetine, a widely prescribed medication for treatment of depression, can affect synaptic plasticity in the adult central nervous system. However, it is not well understood whether acute fluoxetine influences synaptic plasticity, especially on hippocampal CA1 long-term depression (LTD), and if so, whether it subsequently impacts hippocampal-dependent spatial memory. Here, we reported that LTD facilitated by elevated-platform stress in hippocampal slices was completely prevented by fluoxetine administration (10 mg/kg, i.p.) 30 min before stress. The LTD was not, however, significantly inhibited by fluoxetine administration immediately after stress. Similarly, fluoxetine incubation (10 μM) during electrophysiological recordings also displayed no influence on the stress-facilitated LTD. In addition, behavioral results showed that a single fluoxetine treatment 30 min before but not after acute stress fully reversed the impairment of spatial memory retrieval in the Morris water maze paradigm. Taken together, these results suggest that acute fluoxetine treatment only before, but not after stress, can prevent hippocampal CA1 LTD and spatial memory retrieval impairment caused by behavioral stress in adult animals.

Fluoxetine, a selective serotonin reuptake inhibitor (SSRI), is widely used to treat major depressive disorder. The antidepressant effect of fluoxetine is mediated by a series of cellular and molecular events, including changes in synaptic plasticity. For example, chronic fluoxetine treatment reinstates ocular dominance plasticity in the primary visual cortex of adult rats, a form of developmentally regulated plasticity that is significantly reduced in the mature brain^1^, and enhances long-term potentiation (LTP) in the dentate gyrus of adult mice^2^. Nonetheless, some studies reported contradictory results that chronic fluoxetine treatment suppresses LTP in the primary auditory cortex^3^ and hippocampus^4^,^5^,^6^ of adult rats. However, all these studies have focused on the potential role of chronic fluoxetine treatment in LTP, whether enhancement or impairment. Correlations between fluoxetine and LTD modulation have not been extensively investigated. One possibility is that LTD is difficult to be induced by classical low frequency stimulation (LFS) protocols in adult animals^7^,^8^,^9^.

It has been well documented that exposure to acute stress impairs hippocampal LTP^6^,^10^,^11^ and facilitates LTD^12^,^13^ in rats, as well as to produce learning and memory impairment in rats and monkeys^8^,^14^,^15^. Previous study has shown that a single systemic injection of fluoxetine is able to reverse the impairment in LTP at synapses from the hippocampus to prefrontal cortex in the rats, caused by stress on an elevated platform^16^,^17^. However, it is not clear whether acute fluoxetine can inhibit acute stress-facilitated LTD in the hippocampus, and if so, it may rescue stress-induced spatial memory retrieval impairment.

In the present study, we investigated the effects of acute fluoxetine treatment on stress-induced hippocampal CA1 LTD and spatial memory retrieval impairment using a combination of *in vitro* electrophysiological and behavioral assessments in adult rats.

## Results

### Single fluoxetine treatment before acute stress inhibits stress-facilitated hippocampal CA1 LTD

**Figure 1:**
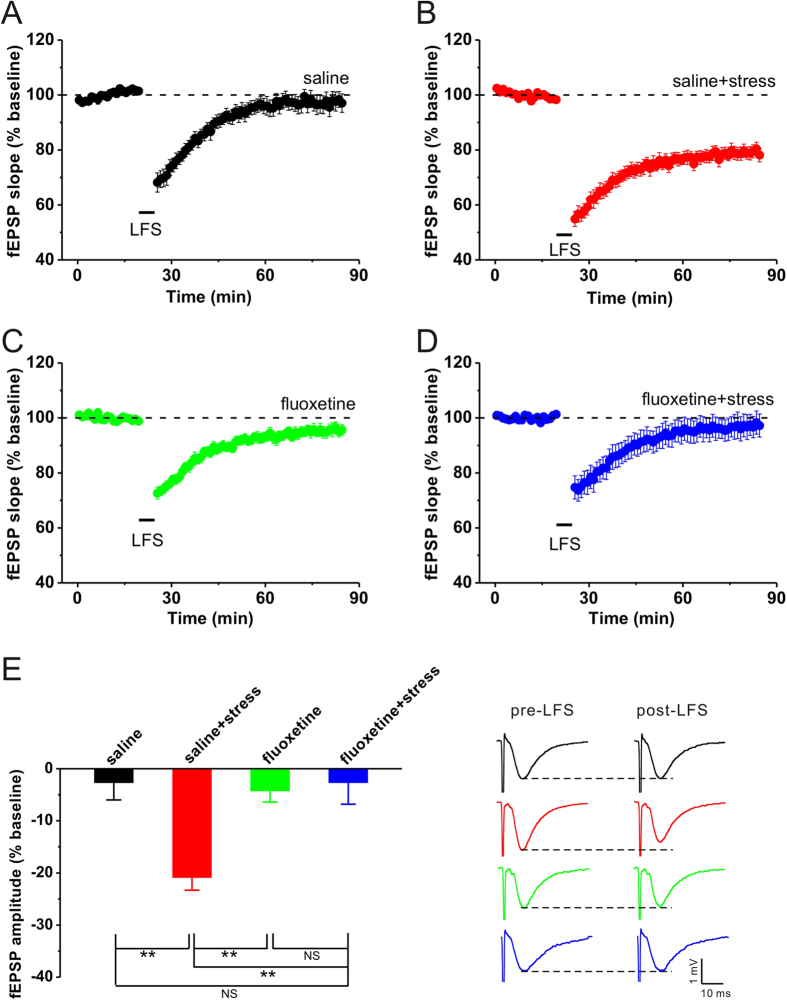
A single injection of fluoxetine before elevated-platform stress prevents stress-facilitated hippocampal CA1 LTD. (**A**) Classical LFS (1 Hz for 15 min) failed to induce LTD in non-stressed control rats. (**B**) Stress enabled LFS to induce a stable LTD in saline-treated rats. (**C**) Fluoxetine (10 mg/kg, i.p.) itself had no effect on LTD induction in non-stressed rats. (**D**) Fluoxetine injection 30 min before stress fully prevented stress-facilitated hippocampal LTD. (**E**) The bar graph summarized the average percentage change of fEPSP amplitude before and 55 min after HFS. Representative traces are shown on the right. **p < 0.01, post hoc Turkey’s test after ANOVA (F_(3, 20)_ = 7.705; p = 0.001).

Previous studies have shown that hippocampal LTD is difficult to induced in adult rats, while acute stress activates the hypothalamus-pituitary-adrenal (HPA) axis, resulting in elevated secretion of corticosterone^15^, and subsequently facilitates LTD production^8^,^12^. Consistent with these results, we found that acute elevated-platform stress dramatically increased plasma corticosterone level compared with control (unstress: n = 6, 94.9 ± 14.6 ng/ml; stress: n = 6, 304.1 ± 31.1 ng/ml, p = 0.001 vs. unstress), and a typical LFS protocol (1 Hz for 15 min) failed to induce hippocampal CA1 LTD in saline control (saline: n = 6, 97.3 ± 3.5%, p = 0.248 vs. baseline; Fig. 1A,E), whereas elevated-platform stress enable LFS to induce a reliable LTD (saline + stress: n = 7, 79.1 ± 2.5%, p < 0.001 vs. baseline, p = 0.004 vs. saline; Fig. 1B,E). Recent studies have reported that fluoxetine can reverse the impairment of LTP caused by stress^16^,^17^. Thus, it is reasonable to assume that fluoxetine may also inhibit hippocampal LTD facilitated by acute stress. As expected, fluoxetine administration (10 mg/kg, i.p.) 30 min before elevated-platform stress succeeded in preventing hippocampal CA1 LTD in stressed rats (fluoxetine + stress: n = 6, 97.3 ± 4.5%, p = 0.454 vs. baseline, p = 0.999 vs. saline, p = 0.010 vs. saline + stress; Fig. 1D,E), while fluoxetine per se had no effect on LTD induction (fluoxetine: n = 5, 96.3 ± 2.6%, p = 0.138 vs. baseline; Fig. 1C,E). These results suggest that pretreatment of fluoxetine can inhibit hippocampal CA1 LTD facilitated by acute behavioral stress.

### Single fluoxetine treatment immediately after acute stress has no effect on stress-facilitated hippocampal CA1 LTD

**Figure 2:**
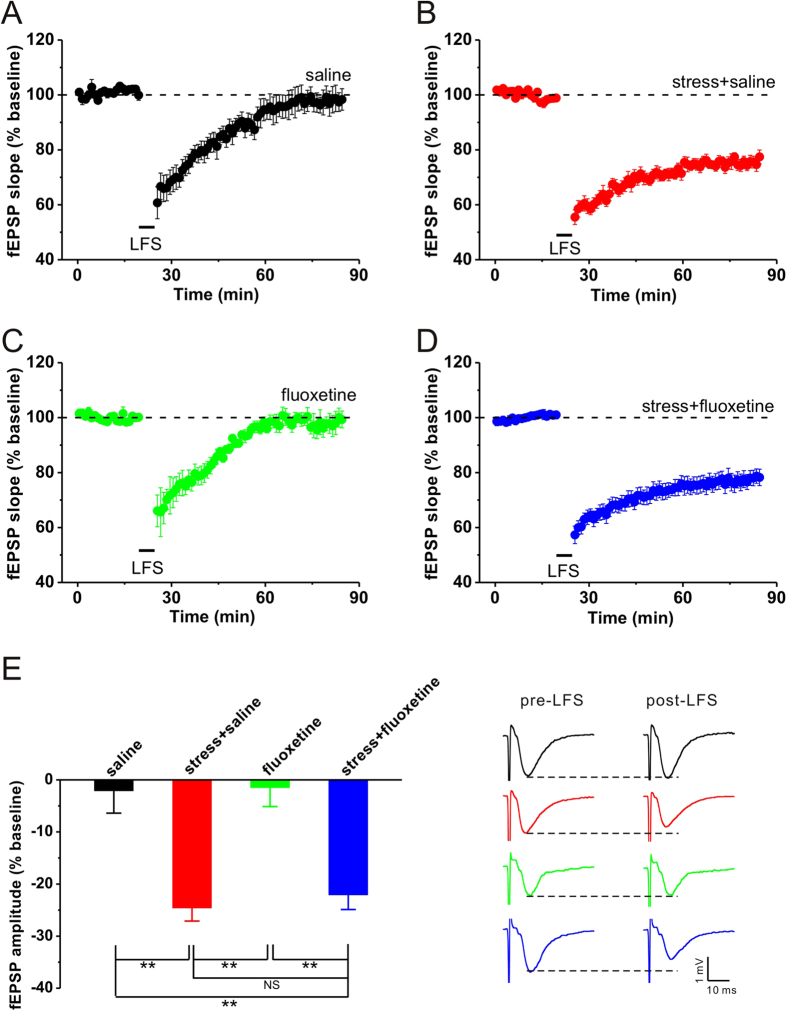
A single injection of fluoxetine immediately after elevated-platform stress fails to prevent stress-facilitated hippocampal CA1 LTD. (**A**) LFS failed to induce hippocampal LTD in non-stressed control. (**B**) Stress enabled LFS to induce a stable LTD in saline-treated rats. (**C**) Fluoxetine (10 mg/kg, i.p.) itself had no effect on LTD induction in non-stressed rats. (**D**) Fluoxetine injection immediately after stress failed to prevent stress-facilitated hippocampal LTD. (**E**) The bar graph summarized the average percentage change of fEPSP amplitude before and 55 min after HFS. Representative traces are shown on the right. **p < 0.01, post hoc Turkey’s test after ANOVA (F_(3, 21)_ = 12.661; p < 0.001).

Next, we wanted to determine whether fluoxetine administration immediately after stress had the same blocking effects on stress-facilitated hippocampal LTD. The results showed that no LTD was induced by LFS in control group (saline: n = 5, 97.9 ± 4.3%, p = 0.347 vs. baseline; Fig. 2A,E), and elevated-platform stress enable LFS to induce a reliable LTD (stress + saline: n = 5, 75.3 ± 2.6%, p = 0.002 vs. baseline, p = 0.002 vs. saline; Fig. 2B,E). Surprisingly, fluoxetine administration immediately after stress failed to prevent hippocampal CA1 LTD in stressed rats (stress + fluoxetine: n = 10, 77.9 ± 2.8%, p < 0.001 vs. baseline, p = 0.002 vs. saline, p = 0.910 vs. stress + saline; Fig. 2D,E), while fluoxetine per se had no effect on LTD induction (fluoxetine: n = 5, 98.5 ± 4.0%, p = 0.799 vs. baseline; Fig. 2C,E). These results suggest that a single treatment with fluoxetine after stress has no effect on hippocampal CA1 LTD facilitated by acute behavioral stress.

### Bath application of fluoxetine has no effect on acute stress-facilitated hippocampal CA1 LTD

**Figure 3:**
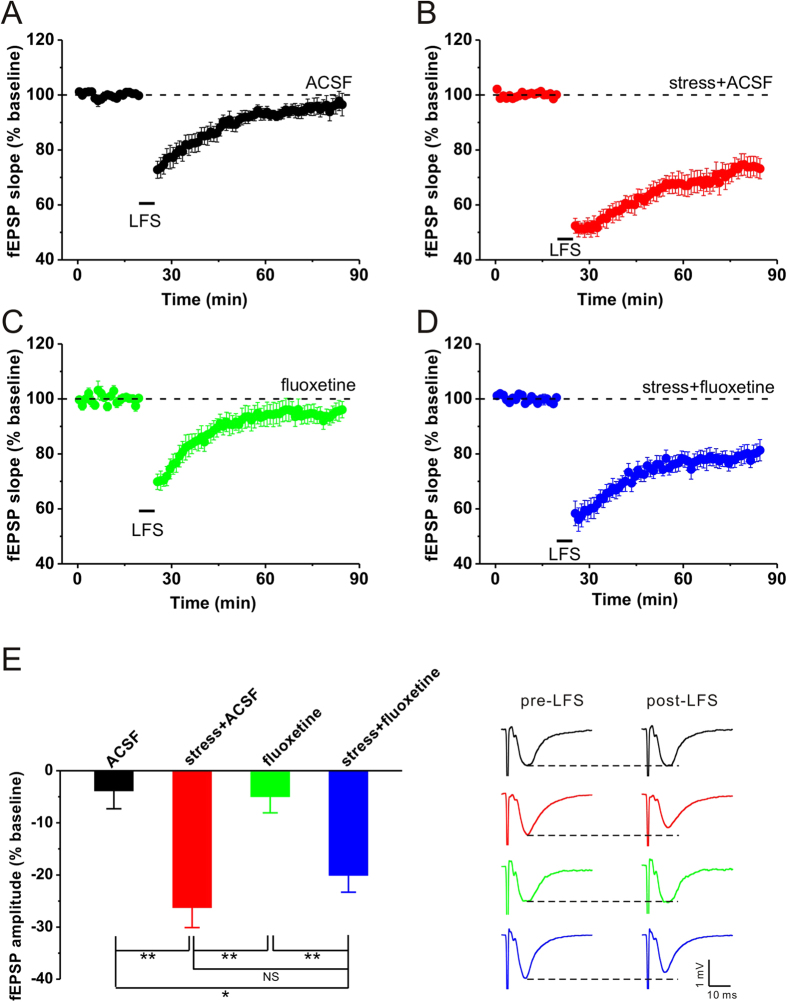
Bath application of fluoxetine during fEPSP recording fails to prevent stress-facilitated hippocampal CA1 LTD. (**A**) LFS failed to induce hippocampal LTD in non-stressed control. (**B**) Stress enabled LFS to induce a stable LTD. (**C**) Fluoxetine (10 μM) itself had no effect on LTD induction in non-stressed rats. (**D**) Bath application of fluoxetine failed to prevent stress-facilitated hippocampal LTD. (**E**) The bar graph summarized the average percentage change of fEPSP amplitude before and 55 min after HFS. Representative traces are shown on the right. *p < 0.05, **p < 0.01, post hoc Turkey’s test after ANOVA (F_(3, 19)_ = 9.954; p < 0.001).

To further confirm the influence of fluoxetine on stress-facilitated hippocampal LTD, we next examined the induction of hippocampal CA1 LTD via direct bath application of fluoxetine during recording. The results showed that no LTD was observed after LFS delivery in ACSF control (ACSF: n = 6, 96.1 ± 3.6%, p = 0.220 vs. baseline; Fig. 3A,E), and elevated-platform stress enable LFS to induce a reliable LTD (stress + ACSF: n = 6, 73.7 ± 3.7%, p < 0.001 vs. baseline, p = 0.002 vs. ACSF; Fig. 3B,E). Similar to fluoxetine administration immediately after behavioral stress, bath application of fluoxetine also failed to prevent hippocampal CA1 LTD in stressed rats (stress + fluoxetine: n = 6, 79.9 ± 2.6%, p = 0.002 vs. baseline, p = 0.018 vs. ACSF, p = 0.996 vs. stress + ACSF; Fig. 3D,E), while fluoxetine per se had no effect on LTD induction (fluoxetine: n = 5, 95.0 ± 3.2%, p = 0.203 vs. baseline; Fig. 3C,E). These results suggest that direct bath application of fluoxetine during recording has no effect on hippocampal CA1 LTD facilitated by acute behavioral stress.

### Single fluoxetine treatment before acute stress prevents the impairment of spatial memory retrieval caused by stress

**Figure 4:**
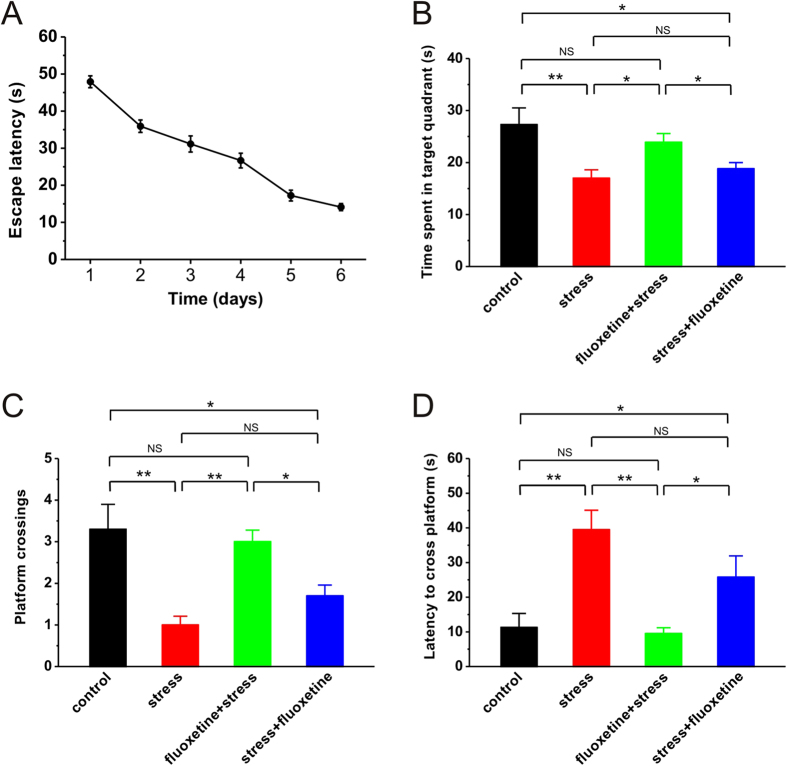
A single injection of fluoxetine before but not after stress reverses the impairment of spatial memory retrieval. (**A**) Plots displayed the average escape latencies of rats for each training day (day 1–6). (**B**) Histograms showed retrieval-test performance of rats from different treatment groups on day 7. Fluoxetine treatment 30 min before but not after stress dramatically rescued the deficit of spatial memory caused by stress, as reflected by spending longer time in the target quadrant where the hidden platform is located during training. *p < 0.05, **p < 0.01, post hoc Turkey’s test after ANOVA (F_(3, 36)_ = 5.734; p = 0.003). (**C**) Histograms summarized the effect of fluoxetine treatment before or after stress on retrieval-test performance, as reflected by hidden-platform crossings. *p < 0.05, **p < 0.01, post hoc Turkey’s test after ANOVA (F_(3, 36)_ = 10.652; p < 0.001). (**D**) Histograms summarized the effect of fluoxetine treatment before or after stress on retrieval-test performance, as reflected by latency of first platform crossing. *p < 0.05, **p < 0.01, post hoc Turkey’s test after ANOVA (F_(3, 36)_ = 9.602; p < 0.001).

Since previous report has shown that hippocampal LTD is necessary and sufficient to mediate acute stress-induced impairment of spatial memory retrieval^8^, it is reasonable to hypothesize that fluoxetine treatment before but not after acute stress may prevent spatial memory retrieval impairment caused by stress. To test this hypothesis, we introduced a widely-used hippocampal-dependent behavioral task, the Morris water maze. In the present experiments, rats were trained to remember the location of a hidden platform over 6 days (Fig. 4A). Twenty-four hours later, their memory for the platform location was tested by using a retrieval trial with the platform absent from the pool. During the retrieval test, stressed rats spent much less time in the target quadrant where the hidden platform was located (stress: n = 10, 17.0 ± 1.6 s, p = 0.005 vs. control; Fig. 4B), compared to non-stressed control (control: n = 8, 27.3 ± 3.2 s, Fig. 4B), confirming the impairment of spatial memory retrieval. As predicted, a single injection of fluoxetine before (fluoxetine + stress: n = 12, 23.9 ± 1.7 s, p = 0.607 vs. control, p = 0.048 vs. stress; Fig. 4B), but not after acute stress (stress + fluoxetine: n = 10, 18.8 ± 1.2 s, p = 0.025 vs. control, p = 0.049 vs. fluoxetine + stress, p = 0.902 vs. stress; Fig. 4B), fully prevented the impairment of spatial memory retrieval. Additionly, the results of crossing the location of hidden platform (control: 3.25 ± 0.56; stress: 1.00 ± 0.21, p < 0.001 vs. control; fluoxetine + stress: 3.00 ± 0.28, p = 0.950 vs. control, p < 0.001 vs. stress; stress + fluoxetine: 1.70 ± 0.26, p = 0.015 vs. control, p = 0.026 vs. fluoxetine + stress, p = 0.430 vs. stress; Fig. 4C) and latency to cross the location of hidden platform (control: 11.3 ± 4.0 s; stress: 39.5 ± 5.6 s, p = 0.001 vs. control; fluoxetine + stress: 9.5 ± 1.7 s, p = 0.992 vs. control, p < 0.001 vs. stress; stress + fluoxetine: 25.8 ± 6.1 s, p = 0.048 vs. control, p = 0.046 vs. fluoxetine + stress, p = 0.164 vs. stress; Fig. 4D) further confirmed that stress impaired spatial memory retrieval, and fluoxetine treatment before, but not after stress could prevent this impairment. Taken together, these results suggest that a single systemic injection of fluoxetine before but not after behavioral stress can prevent the stress-induced impairment of spatial memory retrieval.

## Discussion

The main findings of the present study are an acute injection of fluoxetine before elevated-platform stress prevented stress-facilitated LTD in the CA1 area of hippocampus. In contrast, fluoxetine injection immediately after stress displayed no influence on the LTD. More importantly, by correspondingly applying fluoxetine before behavioral stress, the impairment of spatial memory retrieval caused by stress can be fully reversed. We have therefore provided evidence that acute fluoxetine may block stress-facilitated hippocampal LTD and subsequently rescue memory retrieval impairment.

It has been well documented that hippocampal LTD is difficult to be induced by classical LFS protocol in adult rats^7^,^8^,^9^. However, as demonstrated previously^8^,^12^ and convincingly replicated here (Figs 1, 2, 3), behavioral stress enable LFS to induce a reliable hippocampal LTD. One possible explanation is that stress-enabled LTD may result from hippocampal glucocorticoid receptor activation^7^,^18^, which leads to an increase in glutamate concentration^19^,^20^ and decrease in γ-amino-butyric acid (GABA) concentration^21^ in several brain areas including hippocampus. The alteration of glutamate and GABA level in the hippocampus may result in a lower threshold for LTD induction after stress. However, acute fluoxetine administration significantly elevates GABA level^22^, and then the threshold for LTD induction may return to physiological level. In the current experiment, thus, no obvious LTD was observed in the hippocampus of stressed rats that were pretreated with fluoxetine (Fig. 1). However, Rubio and colleagues have recently reported that a single dose of fluoxetine (0.7 mg/kg, i.p.) administrated 24 h before LFS delivery had no effect on LTD induction at CA3-CA1 synapses^4^. Two important factors in explaining these contradictory findings may be the concentrations and administration time of fluoxetine used to prevent LTD induction. Notably, fluoxetine treatment after behavioral stress had no significant influence on hippocampal LTD production in the present study (Figs 2, 3), which suggested that fluoxetine may exert its actions before but not after the increase in corticosterone secretion. Thus, further experiments need to be performed to determine the exact mechanism underlying different effects of fluoxetine application before or after stress on the restoration of synaptic plasticity in the hippocampus. Alternatively, recent studies show that the chronic effects of antidepressant agents including fluoxetine, are involved in the regulation of intracellular transduction pathways, implicating changes in the cyclic adenosine monophosphate (cAMP) second messenger system, cAMP response element binding protein (CREB) and brain-derived neurotrophic factor (BDNF) in antidepressant action^23^. Given that all these factors have been suggested to contribute to synaptic plasticity^24^,^25^, future studies examining the activities of these transduction pathways and neurotrophic factors after acute fluoxetine treatment will help determine whether the prevention effect of fluoxetine on stress-facilitated LTD can be attributed to its modulation role in these factors.

Hippocampal LTD has been proposed to play a critical role in spatial learning and memory, especially in memory retrieval^8^. Indeed, we here confirmed that stress facilitated hippocampal LTD and subsequently induced a dramatic impairment of spatial memory retrieval (Fig. 4). By blocking hippocampal LTD caused by stress with pretreatment of fluoxetine, the impairment of spatial memory retrieval was fully reversed (Fig. 4). It is noteworthy that pretreatment of fluoxetine not only blocked stress-enabled LTD (Fig. 1) but also prevented the stress-induced impairment of LTP^16^,^17^. Given the previous conjecture that the balance between LTP and LTD may be necessary for learning and memory, it cannot occlude the possibility that normalization of hippocampal LTP after pretreatment of fluoxetine also plays a critical role in preventing the impairment of spatial memory retrieval caused by stress.

In summary, the present work shows that an acute injection of fluoxetine before, but not after elevated-platform stress, reverses the disruption of spatial memory retrieval caused by stress. Furthermore, these behavioral changes are accompanied by the prevention of stress-facilitated LTD in the hippocampus. Thus, these results suggest that acute fluoxetine may serve as a potential therapeutic agent against stress-related psychiatric disorders besides chronic use in major depressive disorder.

## Methods

### Animals

Adult male Sprague-Dawley rats (200–250 g) were obtained from Chongqing Medical University Animal House Center) and were maintained at Children’s Hospital of Chongqing Medical University Animal Care Centre in accordance with the guidelines set forth by Chongqing Medical University Animal Care and Use Committee. Animals were pair-housed in plastic cages in a temperature-controlled (21 °C) colony room on a 12/12 h light/dark cycle. Food and water were available ad libitum. All experiment protocols were approved by Chongqing Medical University Animal Care and Use Committee. All efforts were made to minimize the number of animals used.

### Drugs and treatment

Fluoxetine was purchased from Sigma–Aldrich. For *in vivo* treatment, it was dissolved in 0.9% sterile saline at 10 mg/ml, while it was dissolved in artificial cerebrospinal fluid (ACSF) at 10 μM for *in vitro* treatment.

### Elevated-platform stress

Behavioral stress protocol was similarly to that described previously^8^,^13^ by placing rats on an elevated Plexiglas platform (1.5 m tall, 21 × 21 cm) in a brightly lit room for 30 min^13^. Some rats were subjected to a single fluoxetine (10 mg/kg, i.p.) or the same volume of sterile saline treatment 30 min before stress, and the other rats were treated with fluoxetine or saline immediately after stress. Electrophysiological recordings or behavioral tests were performed 1 h after stress.

### Plasma levels of corticosterone

During the rats were decapitated for slice preparation, blood samples were taken and centrifuged and the serum was stored at −20 °C. Plasma corticosterone levels were determined by using a commercial corticosterone ELISA kit according to the instructions of the manufacturer (Enzo Life Sciences).

### Slice Preparation and Electrophysiology

Slices were prepared by using techniques similar to those described previously^26^,^27^. In brief, rats were deeply anesthetized using urethane (1.5 g/kg, i.p.) and transcardially perfused with NMDG ACSF prior to decapitation. NMDG ACSF contained (in mM): NMDG 93, HCl 93, KCl 2.5, NaH2PO4 1.2, CaCl2 0.5, MgSO4 10, NaHCO3 30, HEPES 20, Na-ascorbate 5.0, Na-pyruvate 3.0, Thiourea 2.0, NAC 12, and D-glucose 25, pH 7.3. Rat brains were rapidly dissected from the skull and placed for sectioning in ice-cold cutting solution (NMDG ACSF) bubbled with 95% O2 and 5% CO2. Coronal hippocampal slices (400 μm thickness) were sectioned from the middle third of hippocampus with a vibratome (VT1000S, Leica Microsystems, Bannockburn, IL) in cutting solution. Slices were then incubated in oxygenated HEPES ACSF for 1 h at 30 °C. HEPES ACSF contained (in mM): NaCl 92, KCl 2.5, NaH2PO4 1.2, CaCl2 0.5, MgSO4 10, NaHCO3 30, HEPES 20, Na-ascorbate 5.0, Na-pyruvate 3.0, Thiourea 2.0, NAC 12, and 25 D-glucose, pH 7.3.

Extracellular recordings were made in the CA1 region of the hippocampus at room temperature (about 25 °C) using a Multiclamp EPC 10 amplifier (HEKA Electronics, Lambrecht/Pfalz, Germany). A bipolar platinum-iridium stimulating electrode was placed among the Schaffer collateral/commissural pathways to elicit field excitatory postsynaptic potentials (fEPSPs), which were recorded from the CA1 stratum radiatum using a glass microelectrode (1 ~ 3 MΩ) filled with 3 M NaCl. The recording electrodes were pulled from borosilicate glass tubing (1.5 mm outer diameter, 0.84 mm inner diameter; World Precision Instruments, Inc.) with a Brown-Flaming micropipette puller (P-97; Sutter Instruments Co.). fEPSPs were evoked by square-wave stimulations (pulse width, 0.1 ms). Test fEPSPs were evoked at a frequency of 0.033 Hz and at a stimulus intensity adjusted to about 50% of the maximal response size. After a 20-min stable baseline, LTD was induced by LFS (900 pulses at 1 Hz).

### Morris water maze test

Spatial learning and memory were examined with the Morris water maze using procedures similar to those described previously^28^,^29^. In brief, the Morris water maze consisted of a circular fiberglass pool (180-cm diameter) filled with water (25 ± 1 °C) that was made opaque with black non-toxic paint. The pool was surrounded by light blue curtains, and three distal visual cues were fixed to the curtains. Four floor light sources of equal power provided uniform illumination to the pool and testing room. A CCD camera suspended above the pool center recorded the swim paths of the animals, and the video output was digitized with an Any-maze tracking system (Stoelting, USA). The pool was artificially divided into four quadrants, i.e., N, E, S, and W. The Morris water maze test included spatial training and a probe test. Twenty-four hours before the spatial training, 40 rats were allowed to adapt to the maze via 60 s of free swimming. The animals were then trained in the spatial learning task for 4 trials per day for 6 consecutive days. In each trial, rats were placed in the water at one of four starting positions (N, E, S, or W) facing to the pool wall. The rats were then required to swim to find the hidden platform (13 cm in diameter, located in the SW quadrant), which was submerged 1 cm under the water. During each trial, the rats were allowed to swim until they found the hidden platform where they remained for 20 s before being returned to a holding cage. The rats that failed to find the hidden platform within 60 s were guided to the platform where they remained for 20 s.

Twenty-four hours after the final training trial, a retrieval test was performed which consisted of a 60-s free swimming period at a novel drop point with the hidden platform absent, and the swimming paths were recorded. During retrieval test, rats were divided into 2 groups: non-stressed control (n = 8) and stressed group (n = 32). To determine the effect of fluoxetine on stress-induced spatial memory retrieval impairment, rats in stressed group were subdivided into 3 subgroups. Twelve rats were subjected to an acute injection of fluoxetine (10 mg/kg, i.p.) 30 min before elevated-platform stress (fluoxetine + stress), and 10 rats were injected with fluoxetine immediately after stress (stress + fluoxetine). The remaining stressed rats were injected with saline vehicle (stress).

### Statistical analysis

All data were expressed as the average percent change from baseline ± SEM and were analyzed by one-way ANOVA followed by post hoc Turkey’s tests where appropriate, with treatment as the between-subjects factor. Significance level was set at p < 0.05.

## Additional Information

**How to cite this article**: Han, H. *et al.* Single fluoxetine treatment before but not after stress prevents stress-induced hippocampal long-term depression and spatial memory retrieval impairment in rats. *Sci. Rep.* **5**, 12667; doi: 10.1038/srep12667 (2015).

## References

[b1] Maya Vetencourt J. F. *et al.* The antidepressant fluoxetine restores plasticity in the adult visual cortex. Science 320, 385–388 (2008). 18420937 10.1126/science.1150516

[b2] Wang J. W., David D. J., Monckton J. E., Battaglia F. & Hen R. Chronic fluoxetine stimulates maturation and synaptic plasticity of adult-born hippocampal granule cells. J Neurosci 28, 1374–1384 (2008). 18256257 10.1523/JNEUROSCI.3632-07.2008 PMC6671574

[b3] Dringenberg H. C., Branfield Day L. R. & Choi D. H. Chronic fluoxetine treatment suppresses plasticity (long-term potentiation) in the mature rodent primary auditory cortex *in vivo*. Neural Plast 2014, 571285 (2014). 24719772 10.1155/2014/571285 PMC3956292

[b4] Rubio F. J. *et al.* Long-term fluoxetine treatment induces input-specific LTP and LTD impairment and structural plasticity in the CA1 hippocampal subfield. Front Cell Neurosci 7, 66 (2013). 23675317 10.3389/fncel.2013.00066 PMC3648695

[b5] Zarei G., Reisi P., Alaei H. & Javanmard S. H. Effects of amitriptyline and fluoxetine on synaptic plasticity in the dentate gyrus of hippocampal formation in rats. Adv Biomed Res 3, 199 (2014). 25337529 10.4103/2277-9175.142044 PMC4202498

[b6] Shakesby A. C., Anwyl R. & Rowan M. J. Overcoming the effects of stress on synaptic plasticity in the intact hippocampus: rapid actions of serotonergic and antidepressant agents. J Neurosci 22, 3638–3644 (2002). 11978839 10.1523/JNEUROSCI.22-09-03638.2002 PMC6758347

[b7] Xu L., Holscher C., Anwyl R. & Rowan M. J. Glucocorticoid receptor and protein/RNA synthesis-dependent mechanisms underlie the control of synaptic plasticity by stress. Proc Natl Acad Sci USA 95, 3204–3208 (1998). 9501241 10.1073/pnas.95.6.3204 PMC19720

[b8] Wong T. P. *et al.* Hippocampal long-term depression mediates acute stress-induced spatial memory retrieval impairment. Proc Natl Acad Sci USA 104, 11471–11476 (2007). 17592137 10.1073/pnas.0702308104 PMC2040922

[b9] Staubli U. & Scafidi J. Studies on long-term depression in area CA1 of the anesthetized and freely moving rat. J Neurosci 17, 4820–4828 (1997). 9169540 10.1523/JNEUROSCI.17-12-04820.1997 PMC6573356

[b10] Diamond D. M., Fleshner M. & Rose G. M. The enhancement of hippocampal primed burst potentiation by dehydroepiandrosterone sulfate (DHEAS) is blocked by psychological stress. Stress 3, 107–121 (1999). 10938573 10.3109/10253899909001116

[b11] Foy M. R., Stanton M. E., Levine S. & Thompson R. F. Behavioral stress impairs long-term potentiation in rodent hippocampus. Behav Neural Biol 48, 138–149 (1987). 2820370 10.1016/s0163-1047(87)90664-9

[b12] Xu L., Anwyl R. & Rowan M. J. Behavioural stress facilitates the induction of long-term depression in the hippocampus. Nature 387, 497–500 (1997). 9168111 10.1038/387497a0

[b13] Dong Z. *et al.* Hippocampal long-term depression mediates spatial reversal learning in the Morris water maze. Neuropharmacology 64, 65–73 (2013). 22732443 10.1016/j.neuropharm.2012.06.027

[b14] Murphy B. L., Arnsten A. F., Jentsch J. D. & Roth R. H. Dopamine and spatial working memory in rats and monkeys: pharmacological reversal of stress-induced impairment. J Neurosci 16, 7768–7775 (1996). 8922432 10.1523/JNEUROSCI.16-23-07768.1996 PMC6579090

[b15] Kim J. J. & Diamond D. M. The stressed hippocampus, synaptic plasticity and lost memories. Nat Rev Neurosci 3, 453–462 (2002). 12042880 10.1038/nrn849

[b16] Rocher C., Spedding M., Munoz C. & Jay T. M. Acute stress-induced changes in hippocampal/prefrontal circuits in rats: effects of antidepressants. Cereb Cortex 14, 224–229 (2004). 14704220 10.1093/cercor/bhg122

[b17] Spennato G., Zerbib C., Mondadori C. & Garcia R. Fluoxetine protects hippocampal plasticity during conditioned fear stress and prevents fear learning potentiation. Psychopharmacology (Berl) 196, 583–589 (2008). 17992518 10.1007/s00213-007-0993-7

[b18] Pavlides C., Kimura A., Magarinos A. M. & McEwen B. S. Hippocampal homosynaptic long-term depression/depotentiation induced by adrenal steroids. Neuroscience 68, 379–385 (1995). 7477947 10.1016/0306-4522(95)94332-s

[b19] Abraham I., Juhasz G., Kekesi K. A. & Kovacs K. J. Corticosterone peak is responsible for stress-induced elevation of glutamate in the hippocampus. Stress 2, 171–181 (1998). 9787265 10.3109/10253899809167281

[b20] Kim S. Y. *et al.* Acute restraint-mediated increases in glutamate levels in the rat brain: an *in vivo* (1)H-MRS study at 4.7 T. Neurochem Res 37, 740–748 (2012). 22187117 10.1007/s11064-011-0668-y

[b21] Briones-Aranda A., Rocha L. & Picazo O. Alterations in GABAergic function following forced swimming stress. Pharmacol Biochem Behav 80, 463–470 (2005). 15740789 10.1016/j.pbb.2005.01.002

[b22] Goren M. Z., Kucukibrahimoglu E., Berkman K. & Terzioglu B. Fluoxetine partly exerts its actions through GABA: a neurochemical evidence. Neurochem Res 32, 1559–1565 (2007). 17486443 10.1007/s11064-007-9357-2

[b23] Duman R. S., Heninger G. R. & Nestler E. J. A molecular and cellular theory of depression. Arch Gen Psychiatry 54, 597–606 (1997). 9236543 10.1001/archpsyc.1997.01830190015002

[b24] Kandel E. R. The molecular biology of memory: cAMP, PKA, CRE, CREB-1, CREB-2, and CPEB. Mol Brain 5, 14 (2012). 22583753 10.1186/1756-6606-5-14 PMC3514210

[b25] Lu B., Nagappan G. & Lu Y. BDNF and synaptic plasticity, cognitive function, and dysfunction. Handb Exp Pharmacol 220, 223–250 (2014). 24668475 10.1007/978-3-642-45106-5_9

[b26] Li H. B. *et al.* Antistress effect of TRPV1 channel on synaptic plasticity and spatial memory. Biol Psychiatry 64, 286–292 (2008). 18405883 10.1016/j.biopsych.2008.02.020

[b27] Wu X. *et al.* Lithium ameliorates autistic-like behaviors induced by neonatal isolation in rats. Front Behav Neurosci 8, 234 (2014). 25018711 10.3389/fnbeh.2014.00234 PMC4071979

[b28] Ge Y. *et al.* Hippocampal long-term depression is required for the consolidation of spatial memory. Proc Natl Acad Sci USA 107, 16697–16702 (2010). 20823230 10.1073/pnas.1008200107 PMC2944752

[b29] Dong Z. *et al.* Long-term potentiation decay and memory loss are mediated by AMPAR endocytosis. J Clin Invest 125, 234–247 (2015). 25437879 10.1172/JCI77888 PMC4382266

